# Supplementary material for: Costs and cost-effectiveness of management of possible serious bacterial infections in young infants in outpatient settings when referral to a hospital was not possible: Results from randomized trials in Africa
Source: PLoS One. 2021 Mar 15;16(3):e0247977. doi: 10.1371/journal.pone.0247977 (PMC7959374; doi:10.1371/journal.pone.0247977)
Supplement: S1 Table — (DOCX) [file pone.0247977.s001.docx]

**S1 Table:** **Health system and management of patients at five study sites**

|  | Democratic Republic of Congo | Kenya | Nigeria (three sites) |
| --- | --- | --- | --- |
| Site | Equateur Province - North and South Ubangi | Western province - Bungoma South, Bungoma East, Bungoma West, Busia, Butula, Teso North, Teso South, Nambale, Bumula, Chwele, Matungu and Mumias districts | Nigeria three sites -Ido and Lagelu local government authorities (LGA), Oyo State, Ile-Ife LGA, Osun State and Zaria LGA, Kadu.na State |
| Study population | 400,000 | 400,000 | 700,000 |
| Pregnancy surveillance and two visits during pregnancy for health promotion and prepare for skill birth attendant delivery | Community health workers (CHW) in the community | CHW in the community | Community health extension workers in the community |
| Identification of births | CHWs through families and other means in the community | CHWs through families and other means in the community | Community health extension workers in the community and traditional birth attendant (TBAs) |
| Approximate Births/year | 17,000 | 13,000 | 20,000 |
| Ten postnatal home visits on day of birth (day 1) and days 3, 7, 14, 21, 28, 35, 42, 49, and 60 of birth | CHWs | CHWs | CHEWs |
| Expected infants with PSBI /year | 2000 | 1500 | 2500 |
| Identification of Sick young infants | CHWs in the community and families | CHWs in the community and families | CHEWs in the community |
| Confirmation of PSBI signs, referral and re-classification if referral refused | Registered nurses at health centers | Registered nurses at health centers | Registered nurses at home of sick young infant |
| Enrolment in the trials | Registered nurses at health centers | Registered nurses at health centers | Registered nurses at home of sick young infant |
| Treatment and follow-up | Registered nurses at health centers after enrolment. Oral antibiotics were given by mothers under supervision of CHWs | Registered nurses at health centers after enrolment. Oral antibiotics were given by mothers under supervision of CHWs | Registered nurses at patients home initiated the therapy after confirmation of diagnosis and enrolment  Thereafter injectable treatment was continued by CHEWs and oral antibiotics were given by mothers under supervision of CHEWs |
