# Supplementary material for: Costs and cost-effectiveness of management of possible serious bacterial infections in young infants in outpatient settings when referral to a hospital was not possible: Results from randomized trials in Africa
Source: PLoS One. 2021 Mar 15;16(3):e0247977. doi: 10.1371/journal.pone.0247977 (PMC7959374; doi:10.1371/journal.pone.0247977)
Supplement: S2 Table — (DOCX) [file pone.0247977.s002.docx]

**S2 Table**: **Quantities of medicines and consumables required for a seven-day treatment under different regimens**

| **Medicines** | | **Quantities per child for a complete 7-day treatment** | | **Consumable types and nos./amount per child per drug use for a 7-day treatment** | |
| --- | --- | --- | --- | --- | --- |
| **Regimen A (reference treatment for both AFRINEST trials)** | | | | |  |
| Gentamicin injection (40 mg/ml) 2 ml ampoule | 1x7=7 | | 2 cc syringes - 7  Needles* – 14  Spirit – 7 ml  Cotton swabs –14  DWVs** – 7  5 cc syringes - 7 (Nigerian sites only) | |  |
| Procaine penicillin injection (1 million units per vial) | 1x7=7 | | 2 cc syringes - 7  Needles* – 14  Spirit – 7 ml  Cotton swabs – 14  DWV – 7  5 cc syringe - 7 (Nigerian sites only) | |  |
| **Regimen B (experimental arm – clinical severe infection)** | | | | |  |
| Oral amoxicillin (250 mg/5 ml) 100 ml bottle | 2x7=14 (full bottle; unused amount discarded) | | 20 cc syringes - 1  5 cc syringes - 1  DWVs – 14 (in Ile-Ife only) | |  |
| Gentamicin injection (40 mg/ml) 2 ml ampoule | 1x7=7 | | 2 cc syringes - 7  Needles – 14  Spirit – 7 ml  Cotton swabs –14  DWVs – 7  5 cc syringes - 7 (Nigerian sites only) | |  |
| **Regimen C (experimental arm – clinical severe infection)** | | | | |  |
| Gentamicin injection (40 mg/ml) 2 ml ampoule | 1x2=2 | | 2 cc syringes - 2  Needles – 4  Spirit – 2 ml  Cotton swabs - 4  DWVs – 2  5 cc syringes - 2 (Nigerian sites only) | |  |
| Procaine penicillin injection (1 million units per vial) | 1x2=2 | | 2 cc syringes – 2  Needles* – 4  Spirit – 2ml  Cotton swabs –4  DWVs – 2  5 cc syringes - 2 (Nigerian sites only) | |  |
| Oral Amoxicillin (250 mg/5 ml) 100 ml bottle | 2x5=10 | | 20 cc syringes-1  5 cc syringes -1  DWVs – 10 (in Ile-Ife only) | |  |
| **Regimen D (experimental arm – clinical severe infection)** | | | | |  |
| Gentamicin injection (40 mg/ml) 2 ml ampoule | 1x2=2 | | 2 cc syringes – 2  Needles – 4  Spirit – 2 ml  Cotton swabs –4  DWVs – 2  5 cc syringes - 2 (Nigerian sites only) | |  |
| Oral amoxicillin (250 mg/5 ml) 100 ml bottle | 2x7=14 (full bottle; unused amount discarded) | | 20 cc syringes - 1  5 cc syringes -1  DWVs – 14 (in Ile-Ife only) | |  |
| **Regimen E (experimental arm for fast breathing only)** | | | | |  |
| Oral amoxicillin (250 mg/5 ml) 100 ml bottle | 2x7=14 (full bottle; unused amount discarded | | 20 cc syringes - 1  5 cc syringes - 1  DWVs – 14 (in Ile-Ife only) | |  |
|  |  | |  | |  |

* In the Nigeria sites, additional needles were used per administration of penicillin and gentamicin: one to draw, second for injecting, and third for dilution. An addition 5 cc syringe was used for dilution. For gentamicin, Ile-Ife used additional DWVs.

** DWV – Distilled water vial
