# Supplementary material for: Costs and cost-effectiveness of management of possible serious bacterial infections in young infants in outpatient settings when referral to a hospital was not possible: Results from randomized trials in Africa
Source: PLoS One. 2021 Mar 15;16(3):e0247977. doi: 10.1371/journal.pone.0247977 (PMC7959374; doi:10.1371/journal.pone.0247977)
Supplement: S3 Table — (DOCX) [file pone.0247977.s003.docx]

**S3 Table: Number of young infants enrolled and treated for the whole period in the AFRINEST studies and 2012 used for costing**

| **Number of Young infants** | **DRC – Equateur province** | **Kenya – Western province** | **Nigeria – Ibadan** | **Nigeria – Ile Ife** | | **Nigeria – Zaria** | |
| --- | --- | --- | --- | --- | --- | --- | --- |
| **Enrolled and treated under the study for the whole period*** | 1006 | 2134 | 952 | 967 | 838 | |  |
| **Enrolled and treated during 2012** | 408 | 646 | 504 | 409 | 343 | |  |

*From April 2011 to June 2013, See [14,15]
