# Supplementary material for: Costs and cost-effectiveness of management of possible serious bacterial infections in young infants in outpatient settings when referral to a hospital was not possible: Results from randomized trials in Africa
Source: PLoS One. 2021 Mar 15;16(3):e0247977. doi: 10.1371/journal.pone.0247977 (PMC7959374; doi:10.1371/journal.pone.0247977)
Supplement: S4 Table — (DOCX) [file pone.0247977.s004.docx]

**S4 Table: Total number of health providers and those surveyed for the costing study at five study sites**

| Provider type | **DRC – Equateur province** | | **Kenya – Western province** | | **Nigeria – Ibadan** | | **Nigeria – Ile Ife** | | **Nigeria – Zaria** | |
| --- | --- | --- | --- | --- | --- | --- | --- | --- | --- | --- |
|  | Total | Surveyed | Total | Surveyed | Total | Surveyed | Total | Surveyed | Total | Surveyed |
| CHW* | 113 | 97 | 154 | 141 |  |  |  |  |  |  |
| CHEW† |  |  |  |  | 32 | 29 | 33 | 28 | 40 | 27 |
| Nurses | 29 | 29 | 67 | 35 | 4 | 4 | 6 | 4 | 8 | 7 |

*CHW – Community Health Worker

† CHEW – Community Health Extension Worker (in Nigerian sites only)

Note: In some cases, the surveyed staff also included the independent outcome assessment nurses or CHW supervisors. In those cases, if the total number of staff surveyed was more than 100%, then only 100% were included.
