# Supplementary material for: Costs and cost-effectiveness of management of possible serious bacterial infections in young infants in outpatient settings when referral to a hospital was not possible: Results from randomized trials in Africa
Source: PLoS One. 2021 Mar 15;16(3):e0247977. doi: 10.1371/journal.pone.0247977 (PMC7959374; doi:10.1371/journal.pone.0247977)
Supplement: S5 Table — (DOCX) [file pone.0247977.s005.docx]

**S5 Table: Price of medicines and consumable supplies by the site (US$)**

|  | International procurement prices (US$) | Democratic Republic of Congo | Kenya | Nigeria | | | | |
| --- | --- | --- | --- | --- | --- | --- | --- | --- |
|  |  |  |  | Ibadan | Ile Ife | | Zaria | |
| **Medicines** | | | | | | | | |
| Oral amoxicillin (powder) (250 mg/5 ml) 100 ml bottle - per treatment | 1.3 | 0.25 | 0.58 | 1.3 | | 1.3 | | 1.3 |
| Gentamicin injection (liquid) (40 mg/ml) - 2 ml ampoule - per administration | 0.173 | 0.14 | 0.23 | 0.173 | | 0.173 | | 0.173 |
| Procaine penicillin (powder) (1 million units vial) – per administration) | 0.444 | 0.37 | 0.35 | 0.444 | | 0.444 | | 0.444 |
|  |  |  |  |  | |  | |  |
| **Consumable supplies** | | | | | | | | |
| Measuring syringe (20 cc) |  | 0.17 | 0.23 | 0.06 | | 0.10 | | 0.13 |
| 5 cc syringe |  | 0.12 | 0.02 | 0.04 | | 0.05 | | 0.10 |
| 2 cc syringe |  | 0.04 | 0.02 | 0.04 | | 0.05 | | 0.06 |
| Needles |  | 0.04 | 0.12 | 0.02 | | 0.02 | | 0.03 |
| Spirit per unit of administration |  | 0.01 | 0.02 | 0.01 | | 0.02 | | 0.02 |
| Cotton swabs per unit of administration |  | 0.01 | 0.06 | 0.01 | | 0.01 | | 0.01 |
| Distilled water vials |  | 0.06 | 0.20 | 0.10 | | 0.05 | | 0.06 |
