# Supplementary material for: Costs and cost-effectiveness of management of possible serious bacterial infections in young infants in outpatient settings when referral to a hospital was not possible: Results from randomized trials in Africa
Source: PLoS One. 2021 Mar 15;16(3):e0247977. doi: 10.1371/journal.pone.0247977 (PMC7959374; doi:10.1371/journal.pone.0247977)
Supplement: Attachment — Submitted filename: PONE-D-20-10269_reviewer.pdf [file pone.0247977.s006.pdf]

PLOS ONE

Costs and cost-effectiveness of management of possible serious bacterial infections in young infants in outpatient settings when referral to hospital was not possible: Results from randomized trials in Africa

--Manuscript Draft--

Manuscript Number:	PONE-D-20-10269
Article Type:	Research Article
Full Title:	Costs and cost-effectiveness of management of possible serious bacterial infections in young infants in outpatient settings when referral to hospital was not possible: Results from randomized trials in Africa
Short Title:	Costing treatment of possible serious bacterial infections in young infants in outpatient settings
Corresponding Author:	Charu C Garg, Ph.D.<br>World Healrh Organization<br>TORONTO, Ontario CANADA
Keywords:	Costs; Cost-effectiveness; Possible Serious Bacterial Infection; clinical severe infections; community management; outpatient treatment; child; young infants; Africa; Kenya; Nigeria; Democratic Republic of Congo
Abstract:	Introduction: Limited information exists about the cost of implementing a strategy of treating young infants up to 59 days of age with signs of possible serious bacterial infection (PSBI) when referral is not feasible.<br>Method: We used data from the African Neonatal Sepsis Trial (AFRINEST), conducted at five sites in three countries (Democratic Republic of Congo [DRC], Kenya, and Nigeria), to determine costs of simplified antibiotic regimens for treatment of PSBI in young infants up to 59 days of age in outpatient settings in order to compare their efficacy to a reference treatment. Direct costs were estimated for human resources for pregnancy and birth surveillance, home visits, diagnosis, treatment and follow-up of PSBI cases for each of five regimens. Indirect costs included training, transport, communication, non-consumables, and programme management. Administrative costs included human resource time for general management, supervision and coordination. The incremental cost-effectiveness ratio (ICER) was calculated using treatment failure after one week as the outcome indicator.<br>Results: The average costs of treating a young infant in the clinical severe infection (CSI) sub-category of PSBI was lowest with injectable gentamicin for two days plus oral amoxicillin for seven days (regimen D) at US$ 28.2 (95% CI US$ 22.7–33.6) compared to injectable gentamicin plus oral amoxicillin for seven days (regimen B) at US$ 32.3 (95% CI US$ 26.5–38.1) and injectable gentamicin plus injectable procaine penicillin for seven days (reference regimen A) at US$ 35.1 (95% CI US$ 28.9–40.3). The ICER showed regimen B to be the most cost-effective option followed by regimen D. For fast breathing, oral amoxicillin for seven days (regimen E) was more cost-effective than regimen A. Indirect and administrative costs, one third to one half of the total treatment costs, were important for effective management of PSBI in outpatient settings.<br>Conclusion: Treatment of PSBI with simplified regimens in outpatient settings when referral is not feasible is cost-effective.
Order of Authors:	Charu C Garg, Ph.D.
	Antoinette Tshefu
	Adrien Lokangaka Longombe
	Jean-Serge Ngaima Kila
	Fabian Esamai
	Peter Gisore
	Adejumoke Idowu Ayede
	Adegoke Gbadegesin Falade
	Ebunoluwa A. Adejuyigbe

Powered by Editorial Manager® and ProduXion Manager® from Aries Systems Corporation

	Chineme Henry Anyabolu
	Robinson D. Wammanda
	Joshua Daba Hyellashelni
	Sachiyo Yoshida
	Lu Gram
	Shamim A Qazi
	Rajiv Bahl
**Additional Information:**	
**Question**	**Response**
**Financial Disclosure**<br><br>Enter a financial disclosure statement that describes the sources of funding for the work included in this submission. Review the submission guidelines for detailed requirements. View published research articles from *PLOS ONE* for specific examples.<br><br>This statement is required for submission and **will appear in the published article** if the submission is accepted. Please make sure it is accurate.<br><br>**Unfunded studies**<br>Enter: *The author(s) received no specific funding for this work.*<br><br>**Funded studies**<br>Enter a statement with the following details:<br>• Initials of the authors who received each award<br>• Grant numbers awarded to each author<br>• The full name of each funder<br>• URL of each funder website<br>• Did the sponsors or funders play any role in the study design, data collection and analysis, decision to publish, or preparation of the manuscript?<br>• **NO** - Include this sentence at the end of your statement: *The funders had no role in study design, data collection and analysis, decision to publish, or preparation of the manuscript.*<br>• **YES** - Specify the role(s) played.<br><br>* typeset	The study was funded by Bill and Melinda Gates Foundation (www.gatesfoundation.org) through a grant number OPPGH5299 to World Health Organization, Geneva, Switzerland.<br><br>AT, FE, AIA, EAA, RDW, received awards from the same BMGF grant OPPGH5299 to conduct field work at 5 sites and collect data. CCG was awarded a consultant contract from WHO to conduct costing analysis.<br><br>The funders had no role in study design, data collection and analysis, decision to publish, or preparation of the manuscript.
**Competing Interests**	The authors have declared that no competing interests exist

Use the instructions below to enter a competing interest statement for this submission. On behalf of all authors, disclose any competing interests that could be perceived to bias this work—acknowledging all financial support and any other relevant financial or non-financial competing interests.<br><br>This statement **will appear in the published article** if the submission is accepted. Please make sure it is accurate. View published research articles from *PLOS ONE* for specific examples.<br><br>**NO authors have competing interests**<br><br>Enter: *The authors have declared that no competing interests exist.*<br><br>**Authors with competing interests**<br><br>Enter competing interest details beginning with this statement:<br><br>*I have read the journal's policy and the authors of this manuscript have the following competing interests: [insert competing interests here]*<br><br>* typeset	
**Ethics Statement**<br><br>Enter an ethics statement for this submission. This statement is required if the study involved:<br><br>• Human participants<br>• Human specimens or tissue<br>• Vertebrate animals or cephalopods<br>• Vertebrate embryos or tissues<br>• Field research<br><br>Write "N/A" if the submission does not require an ethics statement.<br><br>General guidance is provided below. Consult the submission guidelines for detailed instructions. **Make sure that all**	The study was approved by the institutional ethics committees of each participating institution and the WHO Ethics Review Committee (Protocol ID NCH09008). Written informed consent was obtained from caregivers for each activity.

information entered here is included in the Methods section of the manuscript.<br><br>**Format for specific study types**<br><br>**Human Subject Research (involving human participants and/or tissue)**<br>• Give the name of the institutional review board or ethics committee that approved the study<br>• Include the approval number and/or a statement indicating approval of this research<br>• Indicate the form of consent obtained (written/oral) or the reason that consent was not obtained (e.g. the data were analyzed anonymously)<br><br>**Animal Research (involving vertebrate animals, embryos or tissues)**<br>• Provide the name of the Institutional Animal Care and Use Committee (IACUC) or other relevant ethics board that reviewed the study protocol, and indicate whether they approved this research or granted a formal waiver of ethical approval<br>• Include an approval number if one was obtained<br>• If the study involved *non-human primates*, add *additional details* about animal welfare and steps taken to ameliorate suffering<br>• If anesthesia, euthanasia, or any kind of animal sacrifice is part of the study, include briefly which substances and/or methods were applied<br><br>**Field Research**<br><br>Include the following details if this study involves the collection of plant, animal, or other materials from a natural setting:<br>• Field permit number<br>• Name of the institution or relevant body that granted permission	
**Data Availability**<br><br>Authors are required to make all data underlying the findings described fully available, without restriction, and from the time of publication. PLOS allows rare exceptions to address legal and ethical	Yes - all data are fully available without restriction

concerns. See the PLOS Data Policy and FAQ for detailed information.<br><br>A Data Availability Statement describing where the data can be found is required at submission. Your answers to this question constitute the Data Availability Statement and **will be published in the article**, if accepted.<br><br>**Important:** Stating 'data available on request from the author' is not sufficient. If your data are only available upon request, select 'No' for the first question and explain your exceptional situation in the text box.<br><br>Do the authors confirm that all data underlying the findings described in their manuscript are fully available without restriction?	
**Describe where the data may be found in full sentences. If you are copying our sample text, replace any instances of XXX with the appropriate details.**<br><br>• If the data are **held or will be held in a public repository**, include URLs, accession numbers or DOIs. If this information will only be available after acceptance, indicate this by ticking the box below. For example: *All XXX files are available from the XXX database (accession number(s) XXX, XXX.*).<br>• If the data are all contained **within the manuscript and/or Supporting Information files**, enter the following: *All relevant data are within the manuscript and its Supporting Information files.*<br>• If neither of these applies but you are able to provide **details of access elsewhere**, with or without limitations, please do so. For example:<br><br>*Data cannot be shared publicly because of [XXX]. Data are available from the XXX Institutional Data Access / Ethics Committee (contact via XXX) for researchers who meet the criteria for access to confidential data.*	Data are all contained within the manuscript and/or Supporting Information files

The data underlying the results presented in the study are available from (include the name of the third party and contact information or URL).<br>• This text is appropriate if the data are owned by a third party and authors do not have permission to share the data.<br><br>* typeset	
Additional data availability information:	

Powered by Editorial Manager® and ProduXion Manager® from Aries Systems Corporation

Manuscript

Click here to download Manuscript Costing Paper-PSBI Africa 8 April 2020.docx

8 April 2020

Costs and cost-effectiveness of management of possible serious bacterial infections in young infants in outpatient settings when referral to hospital was not possible: Results from randomized trials in Africa

Short title: Costing treatment of possible serious bacterial infections in young infants

Charu C. Garg[1*¶#a], Antoinette Tshefu[2&], Adrien Lokangaka Longombe [2&], Jean-Serge Ngaima Kila [2&], Fabian Esamai[3&], Peter Gisore[3&], Adejumoke Idowu Ayede[4&], Adegoke Gbadegesin Falade[4&], Ebunoluwa A. Adejuyigbe[5&], Chineme Henry Anyabolu[5&], Robinson D. Wammanda[6&], Joshua Daba Hyellashelni [6&], Sachiyo Yoshida[7&], Lu Gram[8&], Shamim Qazi[1¶,] Rajiv Bahl[7¶], on behalf of the African Neonatal Sepsis Trial (AFRINEST) group

[1] Consultant, Department of Maternal, Newborn, Child and Adolescent Health, World Health Organization, Geneva, Switzerland

[2]Department of Community Health, Kinshasa School of Public Health, Kinshasa, Democratic Republic of Congo

[3]Department of Child Health and Paediatrics, School of Medicine, Moi University, Eldoret, Kenya

[4] Department of Paediatrics, College of Medicine, University of Ibadan, and University College Hospital, Ibadan, Nigeria

[5] Department of Paediatrics and Child Health, Obafemi Awolowo University, Ile-Ife, Nigeria

[6] Department of Paediatrics, Ahmadu Bello University Teaching Hospital, Ahmadu Bello University, Zaria, Nigeria

[7] Department of Maternal, Newborn, Child and Adolescent Health and Ageing, World Health Organization, Geneva, Switzerland

[8] University College London, London, United Kingdom

#a Independent International Consultant, Toronto, Canada,

*** Corresponding author**

Email: charucgarg@gmail.com (CCG)

¶ These authors contributed equally to the work

& These authors also contributed equally to this work

Abstract

Introduction: Limited information exists about the cost of implementing a strategy of treating young infants up to 59 days of age with signs of possible serious bacterial infection (PSBI) when referral is not feasible.

Method: We used data from the African Neonatal Sepsis Trial (AFRINEST), conducted at five sites in three countries (Democratic Republic of Congo [DRC], Kenya, and Nigeria), to determine costs of simplified antibiotic regimens for treatment of PSBI in young infants up to 59 days of age in outpatient settings in order to compare their efficacy to a reference treatment. Direct costs were estimated for human resources for pregnancy and birth surveillance, home visits, diagnosis, treatment and follow-up of PSBI cases for each of five regimens. Indirect costs included training, transport, communication, non-consumables, and programme management. Administrative costs included human resource time for general management, supervision and coordination. The incremental cost-effectiveness ratio (ICER) was calculated using treatment failure after one week as the outcome indicator.

Results: The average costs of treating a young infant in the clinical severe infection (CSI) sub-category of PSBI was lowest with injectable gentamicin for two days plus oral amoxicillin for seven days (regimen D) at US$ 28.2 (95% CI US$ 22.7–33.6) compared to injectable gentamicin plus oral amoxicillin for seven days (regimen B) at US$ 32.3 (95% CI US$ 26.5–38.1) and injectable gentamicin plus injectable procaine penicillin for seven days (reference regimen A) at US$ 35.1 (95% CI US$ 28.9–40.3). The ICER showed regimen B to be the most cost-effective option

followed by regimen D. For fast breathing, oral amoxicillin for seven days (regimen E) was more cost-effective than regimen A. Indirect and administrative costs, one third to one half of the total treatment costs, were important for effective management of PSBI in outpatient settings.

Conclusion: Treatment of PSBI with simplified regimens in outpatient settings when referral is not feasible is cost-effective.

Introduction

Of the 5.3 million estimated child deaths in 2018, 2.5 million occurred in the neonatal period [1], with infections, intrapartum complications and preterm birth accounting for most. In 2016, infections including pneumonia, sepsis and meningitis were responsible for nearly 550 000 (21%) neonatal deaths, nearly all of them occurring in developing countries[2]. The World Health Organization (WHO) recommends postnatal home visits to increase coverage of care and improve newborn survival through identifying sick young infants, promoting appropriate care-seeking and early management. The proportion of deliveries at health facilities is increasing, but in many settings mothers and newborns are discharged within a few hours of birth and have little contact with a health provider until the 6-week postpartum and immunization visit. Births at home pose an even greater challenge for providing care during the first critical hours and days [3-4].

Based on a conservative estimate of 355 500–605 750 annual cases and 177 500–302 870 annual deaths due to neonatal sepsis in sub-Saharan Africa , 5.29–8.73 million disability-adjusted life years (DALYs) are lost each year, leading to an annual economic burden ranging from US$ 10 billion to US$ 469 billion. [5] WHO recommends referral to hospital and injectable therapy for management of PSBI in neonates (0–28 days old) and young infants (0–59 days old) [6-7]. However, in resource-limited settings 60-80% of the families of young infants with signs of severe infection do not accept referral to hospital due to reasons such as distance to the health facility, cost of hospitalization and cultural constraints, resulting in newborn deaths [8-15]. Four randomized controlled trials were conducted in five countries in Africa and Asia to evaluate the safety and effectiveness of simplified antibiotic regimens that could be given on an outpatient

basis for treating PSBI in young infants when referral is not feasible [16- 19]. In the two Asian trials in Bangladesh and Pakistan two simpler alternative antibiotic regimens were compared against a reference regimen, whereas in the African multicentre trial in five sites in three countries (DRC, Kenya and Nigeria), two additional regimens were evaluated (Box 1). These trials showed that simplified antibiotic regimens requiring fewer injections were equivalent in treatment outcomes to the reference regimen for young infants with signs of CSI and fast breathing without signs of critical illness when referral was not feasible [13-16]. The implementation of these findings will increase access to treatment for young infants with PSBI [20]. Limited evidence is available for the costs of PSBI and pneumonia management from low- and middle-income countries (LMICs) [21,22].

We report costs and cost-effectiveness analysis for outpatient treatment of PSBI with simplified antibiotic regimens in three African countries when referral is not feasible.

Methods

AFRINEST was conducted in a total of five sites in DRC (North and South Ubangi), Kenya (Eldoret) and Nigeria (Ile Ife, Ibadan and Zaria) to treat PSBI in neonates and young infants on an outpatient basis when referral was not feasible [13-14]. Five simplified antibiotic regimens were used (see Box 1). Pregnancy and birth surveillance and home visits were conducted. These sites were mainly rural, with some semi-urban and peri-urban areas. The study was conducted from April 2011 to June 2013, with the costs estimated for 2012. Details of the trial methodology have been reported elsewhere [23-24].

Box 1. Description of antibiotic regimens

Reference treatment:

1. Treatment regimen A The reference group received a gentamicin injection once daily and a procaine penicillin injection once daily for 7 days (14 injections in total) (as used in the AFRINEST and Simplified Antibiotic Therapy Trial [SATT] studies) [13-16].

Experimental treatments (intervention):

CSI: a young infant from 0–59 days of age presenting with any of these signs: severe chest indrawing, body temperature ≥ 38.0 °C or < 35.5 °C, stopped feeding well, or movement only when stimulated.

2. Treatment regimen B: gentamicin injection once daily and oral amoxicillin twice daily for 7 days (7 injections in total) (as used in the AFRINEST and SATT studies) [13-16].

3. Treatment regimen C: gentamicin injection once daily and procaine penicillin injection once daily for 2 days, thereafter oral amoxicillin for 5 days (4 injections in total) (as used in the AFRINEST and SATT studies) [13-16].

4. Treatment regimen D: gentamicin injection once daily and oral amoxicillin twice daily for 2 days, thereafter oral amoxicillin twice daily for 5 days (2 injections in total) (as used only in the AFRINEST study) [13].

Fast breathing pneumonia: A young infant from 0-59 days of age presenting with respiratory rate of 60 breaths or more per minute.

5. Treatment regimen E: oral amoxicillin twice daily for 7 days (as used only in the AFRINEST study) [14].

The direct costs of drugs, supplies and medical staff time for different types of services and by different providers were calculated for each mother-child dyad served. Indirect and administrative costs, such as for supervision, training, quality assurance, monitoring and evaluation, non-consumables and operational expenses did not change for every additional child served [25-26]. Some of the costs were considered as research and not included for costing, such as: development of material; initial training of staff; additional equipment and infrastructure; staff hired to monitor the effectiveness of the antibiotic regimens; baseline surveys; workshops, communication equipment and vehicles purchased. The cost of existing capacity, except for health workers' time, were also not included, but activities needed on a recurrent basis, such as refresher training, administration, monitoring meetings, communications and travel for screening were included. There were also non-consumable marginal costs, such as those for weighing scales, thermometers and timers for screening and diagnosis.

The effectiveness indicator was calculated using the risk difference in treatment failure and was estimated as the percentage of newborns who did not fail treatment after one week of enrollment with each of the regimens. Treatment failure was defined as any of the following: death; clinical deterioration or admission to hospital at any time after enrollment; persistence of fast breathing on day 4 or recurrence after day 4 up to day 8; and development of a serious adverse event related to the study antibiotics, such as organ failure, anaphylactic reaction, severe diarrhoea, or severe rash [13-14].

Ethical approvals

The study was approved by the institutional ethics committees of each participating institution and the WHO Ethics Review Committee (Protocol ID NCH09008). Written informed consent was obtained from caregivers for each activity.

Interventions/major activities and sub-activities

The interventions for which costs were derived were only linked to health services delivery in community and outpatient settings. The interventions were classified into four main categories and described below.

1. **Home-based care** (Intervention 1.0): Community Health Workers (CHWs)/ Community Health Extension Workers (CHEWs) carried out community-based surveillance to identify pregnant women and infants who were 0 – 59 days of age (sub-activity 1.1). Thereafter, the CHWs/CHEWs made two antenatal visits for health promotion, such as exclusive breastfeeding for the first 6 months, seeking care from skilled birth attendants during pregnancy and delivery, preparation for delivery, prevention of malaria and promoting good dietary habits (sub-activity 1.2). Only 10% of the costs of these activities was considered important for PSBI management. A further 10 postnatal home visits were made on days 1, 3, 7, 14, 21, 28, 35, 42, 49 and 56 for birth identification and to promote optimal care practices such as breastfeeding, keeping the baby warm and hygiene; to identify danger signs in mothers and newborns; and to promote appropriate care-seeking (sub-activity 1.3). We included 20% of these costs as PSBI treatment and management, and calculated the human resource cost for this intervention based on the time spent by providers. Failed visits where

the provider could not make contact with the caregiver and waiting time were taken into account while calculating average time for two antenatal and 10 postnatal visits. Medicines and consumables were not considered under this intervention.

2. **Link between CHWs and nurses** (Intervention 2.0): Once a CHW/CHEW identified a danger sign, the infant was referred either to a hospital or to the study nurse for management (sub-activity 2.1). The sick young infants were taken to a health facility by CHWs or visited by the nurse at home. CHWs/CHEWs revisited the homes to check the outcome of the referral or treatment (sub-activity 2.2). Only the human resource costs for this intervention were estimated.

3. **Assessment and management of sick young infants using Integrated Management of Childhood Illness (IMCI)** (Intervention 3.0)**:** The nurse assessed the sick young infants , either brought directly by the mother/caregiver or through the CHW/CHEW (sub-activity 3.1). All young infants who had any sign of PSBI were referred to a hospital for further treatment (sub-activity 3.2).

4. **Outpatient treatment** (Intervention 4.0)**:** If the family refused referral, young infants with PSBI were re-classified into three categories. All young infants classified as having CSI were enrolled in the trial after consent was obtained, and randomized to either the reference therapy (regimen A) or one of the experimental treatment regimens (B, C or D) (Box 1) for outpatient treatment (sub-activities 4.2 to 4.5). Young infants with only fast breathing whose families refused referral were classified as having pneumonia, and after obtaining consent, were randomized to either regimen A or oral amoxicillin (regimen E) (Box 1) for outpatient

treatment (sub-activity 4.6). Critically ill young infants with signs such as unconsciousness, convulsions, unable to feed at all, apnea, unable to cry, cyanosis, bulging fontanel, persistent vomiting (defined as vomiting following three attempts to feed the baby within 30 minutes) and weight < 1500 g at the time of presentation were referred to a hospital and not enrolled in the study. All enrolled young infants were assessed daily (sub-activity 4.1). In the research setting, independent outcome assessors, who were experienced nurses, visited the homes of enrolled young infants on days 4, 8, 11 and 15. However, in the government setting only one visit was undertaken by the CHW/CHEW, and those costs were included under 2.2. If treatment failed, the young infant was referred to the hospital. If referral was refused by the family, rescue treatment with injectable ceftriaxone for seven days was given by independent outcome assessors as outreach. The costs of rescue treatment have been excluded.

Outpatient services by a nurse for sick young infants, such as administering injectables or assessment of non-response to treatment, was provided at government clinics in the DRC and Kenya. In about 10-15% of cases, a nurse made a home visit and administered the indicated injections. CHWs supervised administration of the first dose of oral amoxicillin every day at the home of the infant, while the second dose was given by the parent. In Nigeria, CHEWs initially identified sick young infants in the community, and referred them to the nurse at the clinic for assessment, enrollment, randomization and provision of the first injectable dose of treatment. Thereafter, the CHEW administered the first dose of oral amoxicillin daily and provided injectable therapy at the home of the child. The second dose of oral amoxicillin was given by the parent.

Data

Data were collected from each site on the number of visits for each sub-activity to identify pregnant women and young infants *covered* and *treated* under each intervention; time spent by different providers for each activity and salary of the provider; and quantities and prices of consumables and non-consumables for implementing the activities.

The number of young infants *covered* was estimated by adding those that had 1, 2, 3, or up to 10 postnatal visit[illegible]*reated* young infants were estimated by adding those who had 1, 2, 3 or up to seven days of treatment for each regimen. For example, under regimen B in Kenya, if five young infants were treated for two days, three young infants for three days, 12 young infants for four days, two young infants for five days and 219 young infants for seven days, then the total number of young infants treated would be 241 (5+3+12+2+219) and visits would be 1610 (2*5)+(3*3)+(4*12)+(5*2)+(7*219). Table [illegible]rovides the number of visits and treated young infants for each activity, and covered young infants for each site.

Table 1: Number of visits and treated young infants for each activity and number of covered young infants at five different sites in DRC, Kenya and Nigeria

**No.**	**Interventions/ activities**	**DRC –** North and South Ubangi		**Kenya - Eldoret**		**Nigeria - Ibadan**		**Nigeria - Ile Ife**		**Nigeria - Zaria**	
		Visits [a]	Treated [c]	Visits	Treated	Visits	Treated	Visits	Treated	Visits	Treated
**1**	**Home-based care**	205470	NA	211399	NA	184491	NA	141194	NA	94424	NA
1.1	Surveillance visits for finding pregnant women	50000	NA	101000	NA	77000	NA	40000	NA	47000	NA
1.2	Antenatal home visits (0-2) for all	30131	NA	19531	NA	18643	NA	8230	NA	9268	NA

	pregnant woman										
1.3	Postnatal home visits (0-10) for newborn care and to identify danger signs	125339	NA	90868	NA	88848	NA	92964	NA	38156	NA
**2**	**Link between CHEW and nurse/hospital**	3067	NA	3020	NA	1612	NA	1337	NA	2737	NA
2.1	Referral and/or accompanying child to the study nurse or hospital	2135	NA	1521	NA	1063	NA	673	NA	1373	NA
2.2	Re-visit home to check outcome of a referral or treatment	932	NA	1499	NA	549	NA	664	NA	1364	NA
**3**	**Assessment and management of sick children using IMCI**	4997	NA	10026	NA	670	NA	489	NA	1415	NA
3.1	Assessment and management of illness	4709	NA	9844	NA	646	NA	460	NA	1341	NA
3.2	Referral to hospital for critical illness (PSBI)	288	NA	182	NA	24	NA	29	NA	74	NA
**4**	**Outpatient treatment for those who refused referral (CSI or fast breathing)**	18263	1420	28180	2093	8536	635	8035	581	6834	549
4.1	Daily assessments of those enrolled and treated	9144	1404	14082	2088	4275	633	4020	580	3404	543
4.2	Treatment with regimen A	3281	513	5442	814	1626	246	1483	215	1289	222
4.3	Treatment with regimen B	1273	188	1610	241	521	77	509	74	347	56
4.4	Treatment with regimen C	1411	206	1581	237	571	83	560	80	321	52
4.5	Treatment with regimen D	1401	209	1560	231	531	78	560	83	367	55
4.6	Treatment with regimen E	1753	304	3905	570	1012	151	903	129	1106	164
	**Total**	**231724**	**1420**	**252608**	**2093**	**195211**	**635**	**150507**	**581**	**105342**	**549**
	**Total covered [b]**	**13952**		**9844**		**10815**		**12441**		**4406**	

Notes: a. Number of visits was calculated by multiplying covered young infants by the number of visits per infant for a given activity; except in 1.1 and 1.2, the data were recorded for each visit to homes.
b. The number of covered young infants was obtained by adding the total number receiving postnatal visits.
c. The number of treated young infants was calculated by adding those who were treated for at least one up to seven days with any regimen.
d. For the cells marked with NA (not applicable), no. of treated infants is not relevant for that given activity and only the no. of visits are required for calculating the costs.

A time and motion study was carried out on average time taken by CHWs, CHEWs and nurses per visit on pre-prepared forms for each activity, as self-recorded and also by an interviewer (who followed the health provider) on a random day to validate the self-recorded data [27]. Time spent on travel, waiting and visits where no contact could be made with the caregiver was recorded separately and split across the sub-activities depending on the purpose of the travel. Personal time was not included. Data on duration (in minutes) were recorded and estimated per visit per woman or child for which the activity was undertaken (Table 2). If during a visit of 20 minutes, four women were surveyed in a household, then five minutes were estimated per visit per woman. If more than one provider was involved in an activity, then the weighted average cost was calculated based on the percentage of total visits covered by a provider for that activity (shown in brackets in Table 2). For example, in Nigeria the first dose of oral amoxicillin is given by the nurse at the clinic and the subsequent six daily doses by a CHEW at home. For a seven-day treatment with two doses per day, 1/7 (14%) was provided by the nurse and 86% was by the CHEW. The second dose was given by the parent each day, and no human resource costs were attributed. Table 2 also shows where the activity took place - in the clinic or as outreach. While the activities in Table 2 correspond to the activities in Table 1, the treatment under different regimens (activities 4.2-4.6) requires a combination of sub-activities (4a-4c in Table 2)

Table 2: Average time taken in minutes for each provider per visit by activity and monthly salaries of the providers at five sites in the DRC, Kenya and Nigeria, 2012

No.	Activities	% time of worker: minutes				
		DRC – North and South Ubangi	Kenya – Eldoret	Nigeria-Ibadan	Nigeria – Ile-Ife	Nigeria – Zaria
1.1	Surveillance visits for identifying pregnant women	Outreach<br>CHW (95%): 79<br>Nurse (5%): 34	Outreach<br>CHW (100%): 44	Outreach<br>CHEW (100%): 33	Outreach<br>CHEW (100%): 39	Outreach<br>CHEW (100%): 35
1.2	Antenatal home visits (0-2) for all pregnant woman	Outreach<br>CHW (100%): 40	Outreach<br>CHW (100%): 26	Outreach<br>CHEW (100%): 26	Outreach<br>CHEW (100%): 19	Outreach<br>CHEW (100%): 17
1.3	Postnatal home visits (0-10) for newborn care and to identify danger signs	Outreach<br>CHW (100%): 33	Outreach<br>CHW (100%): 26	Outreach<br>CHEW (100%): 29	Outreach<br>CHEW (100%): 24	Outreach<br>CHEW (100%): 16
2.1	Referral and/or accompanying child to study nurse or hospital	Outreach<br>CHW (100%): 65	Outreach<br>CHW (100%): 49	Outreach<br>CHEW (100%): 10	Outreach<br>CHEW (100%): 6	Outreach<br>CHEW (100%): 10
2.2	Re-visit home to check outcome of referral or treatment	Outreach<br>CHW (100%): 38	Outreach<br>CHW (100%): 37	Outreach<br>CHEW (100%): 24	Outreach<br>CHEW (100%): 19	Outreach<br>CHEW (100%): 22
3.1	Assessment and management of illness	Nurse outreach (10%): 131<br>Nurse clinic (90%): 35	Nurse clinic (100%): 30	Nurse clinic (100%): 42	Nurse clinic (100%): 25	Nurse clinic (100%): 17
3.2	Referral to hospital for critical illness (PSBI)	Nurse outreach (10%): 111<br>Nurse clinic (90%): 15	Nurse outreach (5%): 47<br>Nurse clinic (95%): 16	Outreach<br>Nurse (100%): 23	Outreach<br>Nurse (100%): 38	Outreach<br>Nurse (100%): 26
4.1	Daily assessments of those enrolled and treated	Nurse outreach (10%): 116<br>Nurse clinic (90%): 20	Nurse outreach (5%): 103<br>Nurse clinic (95%): 16	Outreach<br>CHEW (100%): 14	Outreach<br>CHEW (100%): 17	Outreach<br>CHEW (100%): 32
Sub-activities required under activities 4.2-4.6 in Table 1						

4a	Administration of oral amoxicillin	CHW outreach (87%): 50<br>Nurse outreach (3%): 110<br>Nurse clinic (10%): 12	CHW outreach (87%): 31<br>Nurse outreach (3%): 64<br>Nurse clinic (10%): 22	CHEW outreach (86%):55<br>Nurse clinic (14%): 12	CHEW outreach (86%):19<br>Nurse clinic (14%): 11	CHEW outreach (86%):43<br>Nurse clinic (14%): 12
4b	Administration of gentamicin injection	Nurse outreach (15%): 100<br>Nurse clinic (85%): 12	Nurse outreach (15%): 54<br>Nurse clinic (85%): 12	CHEW outreach (86%): 35<br>Nurse clinic (14%): 14	CHEW outreach (86%): 20<br>Nurse clinic (14%): 11	CHEW Outreach (86%): 38<br>Nurse clinic (14%):15
4c	Administration of procaine penicillin injection	Nurse clinic (100%): 10	Nurse outreach (10%): 50<br>Nurse clinic (85%): 8	CHEW outreach (86%): 23<br>Nurse clinic (14%): 15	CHEW outreach (86%): 16<br>Nurse clinic (14%): 4	CHEW outreach (86%): 39<br>Nurse outreach (14%): 5
Monthly salary of CHWs/CHEWs (US$)		21	41	115	128	151
Monthly salary of nurses (US$)		200	344	256	224	272

Total human resource costs for each activity and regimen were determined based on the salary of the providers (Table 2) and total duration spent for a visit and an episode (estimated based on times per day and days per child) of treatment.

Data on quantities of drugs and consumables were collected for each regimen. While the injections were per administration, oral amoxicillin was calculated per treatment course. As unused amounts after dilution were not used later, the quantities for treatment with oral amoxicillin remained the same with 14, 10 or 2 doses under different regimens. Based on the number of administrations per day and number of days of treatment, total amounts of drugs and

consumables required for a full seven-day treatment under different regimens are shown in S1 Table.

The price of drugs and consumables was also calculated per dose and per full (seven days) or partial treatment (assumed to be three days). Drugs were centrally provided through WHO, and international procurement prices were used when local prices were not available. In the DRC and Kenya local generic prices were used while international procurement prices were used for Nigeria. Local prices for all consumables were available. The prices of drugs and consumables used across different sites are given in S2 Table.

Costs under various activities and regimens were calculated per young infant treated by adding the per visit cost for human resources, drugs and consumables for a complete seven-day treatment and for an incomplete three-day treatment. Incomplete/failed treatment happened either because the child did not respond, was withdrawn from treatment, or died on any of the days of treatment. The total costs were calculated by adding per visit cost for human resources, drugs and consumables for different interventions and multiplying by the number of children receiving them. We have not included the costs of rescue treatment in our analysis.

Administrative and indirect costs necessary for effective programme implementation were estimated for:

1. Non-consumables: The annualized costs of items such as weighing scales, thermometers and respiratory rate timers were included. International prices were used, and numbers were calculated based on the number of CHWs/CHEWS and nurses with an additional 10% for breakage. Other items used (e.g. calendars, umbrellas, mattresses, kidney trays, safety boxes,

medicine boxes/bags, calibration instruments) were classified as research or health system costs and not included.

2. Personnel costs for administration and supervision: Costs were estimated for a programme manager (10% of time once a week); one supervisor per 5000 population covered (10% of time every day); and all nurses and CHWs/CHEWs for administration and review meetings (10% of time for 50% of days in a month). Estimates were based on full-time equivalents of each staff type (calculated by multiplying the number of staff with the average time spent in a day and the number of days spent for project activities) and their salaries.

3. Infrastructure and equipment: Equipment for power, computing/office, transport and communication were considered as health system costs and not included.

4. Operational costs: Only 10% of the operational costs of transport, communication (airtime, internet and mobile allowances), and meetings with programme managers, nurses and CHWs/CHEWS were included. At least one monthly meeting in each community for all health workers was assumed. The balance of 90% was incurred for research, such as set-up costs of purchasing vehicles and office equipment or maintaining health systems, including utilities (such as water and electricity bills).

5. Training: Costs were estimated for one refresher training course annually for all nurses, CHWs/CHEWS and supervisors considered essential for running the programme effectively. Training of trainers of nurses and CHWs/CHEWS, initial training of CHEWS and nurses, and other costs such as files, training material, and job aids were considered as research costs.

Results

Cost estimation for regimens

The costs per child treated were determined for five regimens and rescue treatment by adding the per child cost of human resources, drugs and consumables. Table 3 presents the disaggregated costs per child for a complete seven days of treatment under each regimen and rescue treatment for each site. The exchange rates used for converting local currency units to US$ 1 for 2012 are 910 Francs (DRC); 86.1 Shillings (Kenya); and 156.15 Naira (Nigeria).

Table 3: Costs of human resources, drugs and consumables per young infant treated for complete seven days treatment under each regimen at five sites in the DRC, Kenya and Nigeria, 2012, US$

Treatment regimen	North and South Ubangi, DRC			Eldoret, Kenya			Ibadan, Nigeria			Ile Ife, Nigeria			Zaria, Nigeria		
	Human resources*	Drugs/ medicines	Consumables	Human resources	Drugs/ medicines	Consumables	Human resources	Drugs/ medicines	Consumables	Human resources	Drugs/ medicines	Consumables	Human resources	Drugs/ medicines	Consumables
A	1.9	3.6	4.1	7.0	4.1	7.5	4.9	4.3	4.5	3.8	4.3	4.4	6.9	4.3	5.5
B	2.2	1.3	2.1	6.0	2.2	4.2	6.7	2.5	3.1	4.1	2.5	3.4	7.3	2.5	3.6
C	1.5	1.3	1.6	3.6	1.7	3.0	4.5	2.5	1.7	2.9	2.5	1.7	5.4	2.5	2.0
D	1.6	0.5	0.9	3.3	1.0	1.5	5.0	1.6	1.1	3.0	1.6	1.2	5.5	1.6	1.3
E	1.3	0.3	0.3	2.2	0.6	0.3	4.3	1.3	0.1	2.6	1.3	0.2	4.8	1.3	0.2

***Human resources across all sites includes the cost of daily assessment of young infants treated.**

The costs of seven days of treatment per young infant treated showed lowest human resource costs in North and South Ubangi, DRC, primarily due to lower salaries and also because all injectables were provided in the clinics so that the travel and waiting time for nurses were

reduced. In Kenya, the regimen that involved injectables administered by a nurse was more expensive due to higher salaries. In Nigeria, CHEWs replaced many functions performed by nurses, such as administering injectables, so the cost of treatment was lower due to lower salaries of CHEWs. However, this lower cost was offset as all treatment except the diagnosis and the first dose in Nigeria was an outreach activity by CHEWs, requiring more time than treatment at a clinic. The drug costs were similar across sites as in most cases international procurement prices were used. However, where generic medicines were used in the DRC and Kenya costs were lower. Kenya had higher costs for consumables (needles and distilled water vials [DWVs]) as compared to all other sites.

The direct costs of treatment for young infants treated for less than seven days were added to those treated for a full seven days to obtain the average cost per child treated for different regimens. The total costs of home-based care (identification, antenatal care, postnatal visits), links to facility, daily assessment and management were calculated based on human resource costs per visit and total number of visits. Only the marginal costs for non-consumables, operations, training and personnel for supervision and administration necessary for running the programme were used. Table 4 shows per child treated and per child covered costs for different treatment regimens including administration.

Table 4: Pre-outpatient, outpatient (OP), indirect/administrative and total costs per treated and covered young infant* for five regimens for PSBI at five sites in the DRC, Kenya and Nigeria, 2012, US$

	DRC	Kenya	**Nigeria:** Ibadan	**Nigeria:** Ile Ife

Treatment with different regimens	Pre-OP treatment costs[1]	OP direct treatment costs[2]	Indirect and administrative costs [3]	Total costs per child = Pre-OP + OP + indirect costs	Pre-OP treatment costs[1]	OP direct treatment costs[2]	Indirect and administrative costs[3]	Total costs per child = Pre-OP + OP + indirect costs	Pre-OP treatment costs[1]	OP direct treatment costs[2]	Indirect and administrative costs[3]	Total costs per child = Pre-OP + OP + indirect costs	Pre-OP treatment costs[1]	OP direct treatment costs[2]	Indirect and administrative costs[3]	Total costs per child =
**Cost per treated child**																
Regimen A	2.7	8.5	13.5	24.7	5.9	17.7	12.7	36.2	13.5	12.7	12.6	38.8	11.8	12.3	14.8	38.[illegible]
Regimen B	2.7	5.4	13.5	21.5	5.9	11.9	12.7	30.5	13.5	11.9	12.6	38.0	11.8	9.8	14.8	36.[illegible]
Regimen C	2.7	4.3	13.5	20.5	5.9	8.3	12.7	26.8	13.5	8.7	12.6	34.8	11.8	7.2	14.8	33.[illegible]
Regimen D	2.7	3.0	13.5	19.1	5.9	5.9	12.7	24.4	13.5	7.6	12.6	33.7	11.8	5.8	14.8	32.[illegible]
Regimen E (for fast breathing)	2.7	1.9	13.5	18.0	5.9	3.1	12.7	21.6	13.5	5.6	12.6	31.7	11.8	4.1	14.8	30.[illegible]
**Costs per covered child**																
Regimen A	0.27	0.36	1.37	2.00	1.25	2.15	2.69	6.09	0.79	0.32	0.74	1.85	0.55	0.25	0.69	1.4[illegible]
Regimen B	0.27	0.13	1.37	1.77	1.25	1.02	2.69	4.96	0.79	0.13	0.74	1.66	0.55	0.11	0.69	1.3[illegible]
Regimen C	0.27	0.12	1.37	1.76	1.25	0.93	2.69	4.87	0.79	0.11	0.74	1.64	0.55	0.10	0.69	1.3[illegible]
Regimen D	0.27	0.10	1.37	1.74	1.25	0.87	2.69	4.81	0.79	0.10	0.74	1.63	0.55	0.09	0.69	1.3[illegible]
Regimen E (for fast breathing)	0.27	0.09	1.37	1.74	1.25	0.88	2.69	4.82	0.79	0.12	0.74	1.65	0.55	0.09	0.69	1.3[illegible]

* *Covered* young infants include all those who had from 1 to 10 postnatal visits. *Treated* young infants were estimated by adding those who had up to 7 days of treatment for each regimen.

[1]Pre-OP treatment costs include home-based care (surveillance, antenatal and postnatal care visits) and links to the facility.

[2]OP direct treatment costs include human resources for daily assessment of newborns and administration of drugs, medicines and consumables.

[3]Indirect and administrative costs include personnel for supervision, management and operation,; training, non-consumables and operations such as transport, communications and meetings.

[4]The total costs may be slightly different from the sums due to rounding errors.

The cost per young infant with clinical severe infection treated with different regimens were highest for reference regimen A (US$ 25 in the DRC to US$ 38 in Nigeria) and lowest for regimen D (US$ 19 in DRC to US$ 32 in Nigeria) at all sites. Treatment for fast breathing alone (regimen

E) varied between US$ 18 in the DRC to US$ 30 in Nigeria compared to US$ 25 - US$ 38 for regimen A. The administrative costs per treated child varied between US$ 12- US$ 16 across sites. Administrative costs were one third of the total treatment costs, except in the DRC, where they were 50% of the total average costs.

The cost of treatment per young infant covered during postnatal visits varied from US$ 2 with regimen A compared to US$ 1.7 with regimen D in the DRC; US$ 6.1 with regimen A and US$ 4.8 with regimen D in Kenya; an average of US$ 2 with regimen A and US$ 1.7 with regimen D in Nigeria. In Kenya the costs per covered young infant were higher as comparatively larger numbers were treated. In Nigeria overall costs of treatment were greater due to higher costs of human resources.

Discussion

Interpretation of results

Our results show that the cost of treating the CSI sub-category of PSBI in young infants when referral was not feasible were lowest with regimen D, compared to the reference treatment. The average costs across all sites for treating young infants with PSBI in outpatient settings with regimen D were US$ 28 (95% CI US$ 22.7–33.6), US$ 29.6 (95% CI US$ 24.4–34.9) with regimen C and US$ 32.3 (95% CI $26.5–$38.1) with regimen B compared to reference regimen A at US$ 35.1 (95% CI US$ 29.9– 40.3), when all costs such as identification, antenatal care, postnatal care, referrals, daily assessments and administrative costs were taken into account. In an average

covered population of 10 292 young infants, investment for outpatient management of clinical severe infection was estimated at an average of US$ 3 (95% CI US$ 1.4–4.7) with regimen A and US$ 2.5 (95% CI US$ 1.3–3.8) with regimen D per covered young infant (where coverage was defined based on young infants receiving up to 10 postnatal visits and varied from 4406 infants in Zaria to 13 952 infants in the DRC).

Our results are comparable to those found in Ethiopia for management of PSBI at health posts, which was US$ 1.78 per 100 000 population in a routine setting with 95% of women receiving at least four visits. The economic costs (including the opportunity costs of the providers) in the Ethiopian study was estimated at US$ 37 (2015 US$), which stated that “adding PSBI management at health post level was estimated to reduce neonatal mortality after day 1 by 17%, translating to a cost per DALY averted of US$ 223 or 47% of GDP per capita, a highly cost-effective intervention by WHO threshold [21].

For fast breathing pneumonia alone, treatment with oral amoxicillin (non-injectable antibiotic) under regimen E, which can be administered by the parent, was more cost-effective than reference regimen A. The average costs with regimen E were US$ 26.2 (95% CI US$20.9–31.5) including identification, antenatal care, postnatal care, referrals, daily assessments and administrative costs across all sites. The corresponding average costs per covered child were US$ 2.6 (95% CI US$1.3–3.9). For management of chest indrawing pneumonia in children in LMICs, Zhang and colleagues reported the cost per episode in 2013 US$ was US$ 4.3 (95% CI US$1.5–8.7) in the community, US$ 51.7 (95% CI US$17.4–91.0) in outpatient facilities and US$ 242.7 (95% CI US$153.6–341.4) to US$ 559.4 (95% CI US$268.9–886.3) at different levels of hospital for inpatient settings [23]. Direct medical costs of chest indrawing pneumonia management from

Pakistan were reported as US$ 1.5 in community ambulatory care and US$ 7.9 in outpatient care in 2013 US dollars.[28] Zhang and colleagues found that the mean length of stay in hospital for children with chest indrawing pneumonia was 5.8 days (IQR 5.3–6.4) in LMICs and 7.7 (IQR 5.5–9.9) in HICs. Outpatient or community treatment is not only beneficial in terms of reduced costs, but is also less disruptive for families and carries less risk of hospital-acquired infections [22]. The reasons for refusal to accept referral to a hospital include lack of permission from concerned family members, lack of child care, religious and cultural beliefs, distance, cost of travel and treatment, concerns around quality of care and attitudes of health workers [13-15].

WHO guideline recommends using regimen B as option 1 and regimen D as option 2 for treatment of clinical severe infection in young infants 0–59 days old when referral is not feasible [20]. Using the risk difference in treatment failures reported in AFRINEST [16-17], and cost per treated child estimated above, the incremental cost-effectiveness ratio for clinical severe infection at average and low rates of treatment failures, regimen B comes out as most cost-effective, followed by regimen D. Compared to reference regimen A, while all three regimens are cost-effective at low and average risk difference, the results at high risk difference show lower cost and lower effect, and therefore need to be evaluated. For fast breathing, treatment with regimen E is more cost-effective than reference regimen A with ICER=-1.1 for average risk difference in treatment failure.

Table 5: Incremental cost-effectiveness ratios for treating young infants with CSI and fast breathing with different regimens for average, low and high-risk difference in treatment failures on day 8 after enrollment

Treatment name	Cost per child treated	Effect average (%)	Effect high	Effect low	ICER average risk difference in outcomes	ICER low risk difference-= high effect	ICER high risk difference= low effect
**Outcome for CSI: % who did not fail treatment in the first week (on day 8) after enrollment**							

Reference regimen A	35.1	91.9	91.9	91.9	0.0	-	-
B	32.3	93.8	96.3	91.8	-1.5	-0.6	28.2
C	29.6	92.5	95.0	89.9	-9.9	-1.8	2.7
D	28.2	94.6	97.0	91.6	-2.6	-1.4	23.1
**Outcome for fast breathing: % who did not fail treatment in the first week (on day 8) after enrollment**							
Reference regimen A	35.1	77.9	77.9	77.9	0.0	0.0	0.0
E	32.3	80.5	83.9	77.1	-1.1	-0.5	3.5

Manandhar and colleagues argued that an intervention that costs less than US$ 127 is cost-effective [29]. Black and colleagues suggested that “interventions costing less than per capita gross national income per DALY averted can be termed “very cost-effective,” and those costing less than three times per capita gross national income can be termed “cost-effective” [30][illegible]he treatment for PSBI costing less than US$ 35 with either regimen used in our study is cost-effective at all sites by these criteria.

Our results show that administrative costs are an important component of managing PSBI on outpatient basis when referral is not feasible. Marginal administrative costs for effective implementation of the programme include management, supervision, meetings between health providers and supervisors, at least one refresher training course annually for all staff delivering services, basic equipment such as weighing scales, thermometers and timers for every nurse and CHW/CHEW, communications and travel. These vary from almost one third to one half of the total treatment costs at different sites, the highest being in the DRC.

Another important component of managing PSBI in outpatient settings is identification of sick young infants, antenatal and postnatal visits including education of the mother. These pre-

enrollment and pre-treatment activities cost more in Nigeria as these are undertaken by CHEWs who are more qualified and paid more than the CHWs in other settings. Daily assessments of treated young infants were considered important for effective outcomes. The cost per child covered and treated also varies depending on the number of visits and per visit costs for the intervention. Larger numbers of visits to households (per child treated) had to be made for finding those with danger signs, especially in Ibadan. In the DRC, even though the salary for the providers was low, the costs increased due to a longer time taken by the health workers to reach the population in communities due to difficult terrain.

Strengths and weaknesses

Our study's strengths were that a randomized controlled trial was used to implement the study, along with standardized training of staff and data collection. The costs captured in research settings are normally higher than in routine work, but have been appropriately allocated with robust assumptions for the government programme. Some items, such as human resources, programmatic and administrative costs are more difficult to estimate in a government setting, where the providers are engaged in more than one activity. Most studies do not estimate and attribute programmatic and administrative costs for effective implementation of the programme. Our study not only estimates these, but also considers pre-treatment costs of actively screening children with danger signs. One potential weakness could be that the study uses the costs data for 2012. In order to make comparisons with recent studies or to use the cost data for advocacy, inflation factor must be applied.

Implications and conclusions

Outpatient management is cost-effective using a combination of injectable plus oral antibiotics for PSBI when referral is not feasible, and oral antibiotic therapy for those with only fast breathing. While the treatment cost for regimen D is slightly lower, regimen B with gentamicin injections once daily and oral amoxicillin twice daily for seven days is more cost-effective for treating clinical severe infection sub-category of PSBI. Identification of pregnant mothers, antenatal and postnatal visits, and identification and management of sick young infants are critical for improving neonatal survival. Community mobilization interventions, strengthening the skills of workers, including CHWs, to identify sick young infants in a timely fashion, empowerment of mothers to recognize sick young infants and prompt care-seeking by families from appropriate health care providers are critical for appropriate management of sick young infants. Follow-up of those treated, and programmatic functions such as training of health workers, communication with families, follow-up of sick infants, supportive supervision of health workers, provision of necessary commodities and correct and prompt administration of treatment are essential for the successful implementation of this intervention.

Acknowledgements

We thank the families who participated in these studies, the project field staff at each site for their hard work and dedication in data collection, and the physicians and administrators at every site who provided data and facilities for the conduct of the study.

References

1. UNICEF, WHO, World Bank, United Nations Population Division. Levels and trends in child mortality: report 2019. Estimates developed by the UN Interagency Group for Child Mortality Estimation. New York: UNICEF; 2019. https://www.who.int/maternal_child_adolescent/documents/levels_trends_child_mortality_2019/en/.

2. UNICEF, WHO, World Bank, United Nations Population Division. Levels and trends in child mortality: report 2018. Estimates developed by the UN Interagency Group for Child Mortality Estimation. New York: UNICEF; 2018. https://childmortality.org/wp-content/uploads/2018/12/UN-IGME-Child-Mortality-Report-2018.pdf.

3. Hodgins S, McPherson R, Kerber K. Postnatal care, with a focus on home visitation: a design decision-aid For policymakers and program managers. Washington DC: USAID, MCSP, Save the Young infants; 2017. https://www.healthynewbornnetwork.org/hnn-content/uploads/PostnatalCarewithaFocusonHomeVisitation.pdf.

4. WHO. WHO recommendations on postnatal care of the mother and newborn. Geneva: WHO; 2013. http://apps.who.int/iris/ bitstream/10665/97603/1/9789241506649_eng.pdf.

5. Ranjeva SL, Warf BC, Schiff SJ. Economic burden of neonatal sepsis in sub-Saharan Africa. BMJ Global Health. 2018;3:e000347. doi:10.1136/ bmjgh-2017-000347.

6. WHO. Integrated Management of Childhood Illness (IMCI), chart booklet. Geneva: WHO; 2014. http://apps.who.int/iris/ bitstream/10665/104772/16/9789241506823_Chartbook_eng.pdf.

7. WHO. Pocket book of hospital care for children: guidelines for the management of common childhood illnesses. 2nd edition. Geneva: WHO; 2013.

8. Bhandari N, Bahl R, Bhatnagar V, Bhan MK. Treating sick young infants in urban slum setting. Lancet. 1996;347:1774–5.

9. Bang AT, Bang RA, Baitule SB, Reddy MH, Deshmukh MD. Effect of home-based neonatal care and management of sepsis on neonatal mortality: field trial in rural India. Lancet. 1999;354:1955–61.

10. Baqui AH, El-Arifeen S, Darmstadt GL, Ahmed S, Williams EK, Seraji HR, et al. Effect of community-based newborn-care intervention package implemented through two service-delivery strategies in Sylhet district, Bangladesh: a cluster randomised controlled trial. Lancet. 2008;371:1936–44.

11. Kumar V, Mohanty S, Kumar A, Misra RP, Santosham M, Awasthi S, et al. Effect of community-based behaviour change management on neonatal mortality in Shivgarh, Uttar Pradesh, India: a cluster-randomised controlled trial. Lancet. 2008;372:1151–62.

12. Zaidi AKM, Tikmani SS, Warraich HJ, Darmstadt GL, Bhutta ZA, Sultana S, et al. Community-based treatment of serious bacterial infections in newborns and young infants: a randomized controlled trial assessing three antibiotic regimens. Pediatr Infect Dis J. 2012;31:667–72

13. Owais A, Sultana S, Stein AD, Bashir NH, Awaldad R, Zaidi AK. Why do families of sick newborns accept hospital care? A community-based cohort study in Karachi, Pakistan. J Perinatol. 2011;31(9):586-92.

14. Kozuki N, Guenther T, Vaz L, Moran A, Soofi SB, Kayemba CN, et al. A systematic review of community-to-facility neonatal referral completion rates in Africa and Asia. BMC Public Health. 2015;15:989.

15. Munthali AC, Mannan H, MacLachlan M, Swartz L, Makupe CM, Chilimampunga C. Non-use of formal health services in Malawi: perceptions from non-users. Malawi Med J. 2014;26(4):126-32.

16. African Neonatal Sepsis Trial (AFRINEST) group, Tshefu A, Lokangaka A, Ngaima S, Engman C, Esamai F, et al. Simplified antibiotic regimens compared with injectable procaine benzylpenicillin plus gentamicin for treatment of neonates and young Infants with clinical signs of severe infection when referral is not possible: a randomized equivalence trial. Lancet. 2015;385:1767–76. http://dx.doi.org/10.1016/S0140-6736(14)62284-4.

17. African Neonatal Sepsis Trial (AFRINEST) group, Tshefu A, Lokangaka A, Ngaima S, Engmann C, Esamai F, et al. Oral amoxicillin compared with injectable procaine benzylpenicillin plus gentamicin for treatment of neonates and young infants with fast breathing when referral is not possible: a randomised, open label, equivalence trial. Lancet. 2015;385:1758–66. http://dx.doi.org/10.1016/S0140-6736(14)62285-6.

18. Baqui A, Saha S, Ahmed A, Shahidulla M, Quasem I, Roth DE, et al. Safety and efficacy of alternative antibiotic regimens compared with 7 day injectable procaine benzylpenicillin and gentamicin for outpatient treatment of neonates and young infants with clinical signs of severe infection when referral is not possible: a randomised open-label, equivalence trial. Lancet Glob Heal. 2015;3:e279–87.
19. Mir F, Nisar I, Tikmani SS, Baloch B, Shakoor S, Jehan F, et al. Simplified antibiotic regimens for treatment of clinical severe infection in the outpatient setting when referral is not possible for young infants in Pakistan (Simplified Antibiotic Therapy Trial [SATT]): a randomised, open-label, equivalence trial. Lancet Glob Health. 2017;5:e177-e185. doi: 10.1016/S2214-109X(16)30335-7.
20. WHO. Guideline: Managing possible serious bacterial infection in young infants when referral is not feasible. Geneva: WHO; 2015. http://www.who.int/maternal_child_adolescent/documents/bacterial-infection-infants/en/**.**
21. Mathews B, Owen H, Sitrin D, Cousens S, Degefie T, Wall S, et al. Community-Based Interventions for Newborns in Ethiopia (COMBINE): cost-effectiveness. analysis. Health Policy Plan. 2017; 32:i21–i32 doi: 10.1093/heapol/czx054.
22. Zhang S, Sammon PM, King I, Ana LA, Andrade L, Toscano CA, et al. Cost of management of severe pneumonia in young children: systematic analysis. J Glob Health. 2016;6(1):010408. doi: 10.7189/jogh.06.010408.

23. African Neonatal Sepsis Trial Group. Treatment of fast breathing in neonates and young infants with oral amoxicillin compared with penicillin-gentamicin combination: study protocol for a randomized, open-label equivalence trial. Pediatr Infect Dis J. 2013; 32 Suppl 1:S33-8. doi:10.1097/INF.0b013e31829ff7eb.

24. African Neonatal Sepsis Trial Group. Simplified regimens for management of neonates and young infants with severe infection when hospital admission is not possible: study protocol for a randomized, open-label equivalence trial. Pediatr Infect Dis J. 2013;32 Suppl 1:S26-32. doi: 10.1097/INF.0b013e31829ff7d1.

25. Wall SN, Mazzeo CI, Adejuyigbe EA, Ayede AI, Bahl R, Baqui AH, et al. Ensuring quality in AFRINEST and SATT: clinical standardization and monitoring. Pediatr Infect Dis J. 2013;32 Suppl 1: S39-45. doi: 10.1097/INF.0b013e31829ff801.

26. Zaidi AK, Baqui AH, Qazi SA, Bahl R, Saha S, Ayede AI, et al. Scientific rationale for study design of community-based simplified antibiotic therapy trials in newborns and young infants with clinically diagnosed severe infections or fast breathing in South Asia and sub-Saharan Africa. Pediatr Infect Dis J. 2013;32 Suppl 1:S7-11. doi: 10.1097/INF.0b013e31829ff5fc.

27. Hendrich A, Chow MP, Skierczynski AB. A 36-hospital time and motion study: How do medical-surgical nurses spend their time? PermJ.2008;12(3):25-34. http://www.ihi.org/Engage/Memberships/Passport/Documents/Hendrich%20-%20Time%20and%20Motion%20Study.pdf.

28. Sadruddin S, Shehzad S, Bari A, Khan A, Khan A, Qazi S. Household costs for treatment of severe pneumonia in Pakistan. Am J Trop Med Hyg. 2012;87:137-43. doi:10.4269/ajtmh.2012.12-0242.

29. Manandhar DS, Osrin D, Shrestha BP, Mesko N, Morrison J, Tumbahangphe KM, et al. Effect of a participatory intervention with women's groups on birth outcomes in Nepal: cluster-randomised controlled trial. Lancet. 2004;364:970-9.

30. Black RE, Laxminarayan R, Temmerman M, Walker N, editors. Reproductive, maternal, newborn, and child health. Disease control priorities, third edition, volume 2. Washington, DC: World Bank; 2016. doi:10.1596/978-1-4648-0348-2.

Supporting information

S1 Table. Quantities of drugs and consumables required for seven day treatment under different regimens

S2 Table. Price of drugs and consumable supplies by site (US$)

supplementary tables

Click here to access/download
Supporting Information
Supplementary tables PLOS costing paper 7 April 2020.docx
