# Supplementary material for: Costs and cost-effectiveness of management of possible serious bacterial infections in young infants in outpatient settings when referral to a hospital was not possible: Results from randomized trials in Africa
Source: PLoS One. 2021 Mar 15;16(3):e0247977. doi: 10.1371/journal.pone.0247977 (PMC7959374; doi:10.1371/journal.pone.0247977)
Supplement: Attachment — Submitted filename: Comments.docx [file pone.0247977.s007.docx]

**Comments by: Kasahun Girma Tareke**

The authors conducted a study on a very important issue and entitled with “Costs and cost-effectiveness of management of possible serious bacterial infections in young infants in outpatient settings when referral to hospital was not possible: Results from randomized trials in Africa.” It was good that the study employed a randomized control trial and was conducted at different settings. Also, the study found important findings that will be used as an input for synthesizing evidence on costs and cost-effectiveness of simplified regimens used to treat PSBI among young infants. However, there are issues on the manuscript. First, the findings represent the context of the study settings that existed before 8 years (2012). Second, findings related to cost of the simplified regimens is predictable. This is because, as injectable regimens are provided as full-treatment regimens, it is obvious that the costs of the drugs and for health care provider’s become increase, which in turn increases the overall cost. Additionally, the manuscript needs major revision before it will be accepted for publication. Therefore, please find below suggestions for strengthening the paper.

**Abstract**

1. Needs serious revision. Introduction: Only focused on costs of implementing PSBI treatment (please add cost-effectiveness). Methods: please include total sample sizes, how data were processed and analyzed. Conclusion: Your conclusion was not consistent with your introduction, objectives and results. It seems that you were compared with in-patient treatment. Please focus to your objectives and results.

**Introduction**

1. Please change ‘Introduction’ to ‘Background’ and make the alignment at middle.
2. Make your reference citation appropriate. E.g. [5] used after the dot; [3-4], [6-7], [13, 14], [23-24], [25-26], etc.
3. Your introduction part lacks logical flow of ideas (i.e., coherence problem) and not written exhaustively.
i. First write the global burden of young infant morbidity and mortality.
ii. Then, describe the global burden of severe bacterial infection, called possible serous bacterial infection, including its definition, in terms of young infant morbidity and mortality with recent data, including your study setting. Why? Please include concepts written from line 11-14 here. This might signify the importance of trial at your study setting.
iii. What are existing recommendations, initiatives and strategies developed and endorsed by the WHO and other concerning bodies to reduce the burden of this infection and attributed deaths among young infant? Hint: please re-phrase from what was written on the introduction part (about PNC; line 4-10) and also add other information like outpatient treatment recommendations, etc.
iv. Define the abbreviations as their first appearance. E.g., PSBI paragraph 2, line 5.
v. Please give some descriptions about the outpatient treatment given to the young infants with PSBI in terms of service coverage and reduced deaths from previous studies.
vi. Please elaborate the type activities conducted during the trial to enhance effectiveness (i.e., think of this is an evaluative research and you should have to provide precise and clear information about the trial).
vii. Who were the first frontline health care providers that provided the treatment service for sick young infants? Who were the supervisors? Please provide the explanation on the role of CHWs/CHEWs and the trained nurse. It is not clear whether CHWs/CHEWs provide treatment service or not? Or who provides the treatment service from the CHWs/CHEWs and trained nurse? Hint: the WHO guideline was developed to provide programmatic and clinical guidance to the CHWs and as per my knowledge the community health workers or health extension workers are the one who provide the service within the community. Additionally, here, give description of the health system of the study settings. For example, there are some countries that provide management of PSBI at home or health post level when referral to hospital is not possible, e.g. Ethiopia. Activities conducted to facilitate management of PSBI among sick young infants when referral to hospital is not possible, for example, provision of trainings, etc. (i.e., think of this is an evaluative research and you should have to provide precise and clear information about the trial).
viii. Please give detail description on the research gap (s)? What is the importance of knowing the costs and cost-effectiveness of the simplified regimen? Therefore, Authors' rationale for conducting this study should be more clearly articulated.

**Method section**

1. What is AFRINEST? Please describe it first.
2. The author’s mentioned that details of the trial methodology have been reported elsewhere [23-24].
i. Description was not provided on your study setting which makes it difficult to follow for readers not familiar with settings Health System. What is the catchment population? How many health facilities are there at which the service is provided at each study settings?
ii. To improve clarity for readers not familiar with your study settings health system, please discuss the qualifications and training of providers responsible for managing PSBI in young infants in the facility and in the community when referral is not possible.
iii. Here, I have tried to read the methods section of reference number 23 and 24, but it was not described clearly where the treatment service provided and what does it mean by outpatient basis. Please re-phrase to make it more informative and describe the health system of the study settings clearly and precisely.
iv. The way you calculated the sample size (i.e., P-value) differs in both studies. Therefore, to which study would the current study belong regarding the sample size issue? Please re-phrase and make informative enough.
v. Please summarize direct, indirect and administrative costs, and non-included costs in table.
3. Please use the PLOS ONE manuscript writing format. You did not put components of method section clearly to make it comprehensive for the reader.
4. The authors mentioned that the direct costs of drugs, supplies and medical staff time for different types of services and by different providers were calculated for each mother-child dyad served. Who were those different health care providers? Do you think that different health care providers (if it was along different professions) assess, classify and treat sick newborns in similar manner or does is result with similar treatment outcome? Hint: you were assessed the cost-effectiveness in terms of treatment failure. Please specify it.
5. **Home-based care** (Intervention 1.0): line 8-9, you mentioned that 10 postnatal home visits were made on days 1, 3, 7, 14, 21, 28, 35, 42, 49 and 56 for…, but on the reference 23 and 24 you mentioned…….49 and 60 for….which lacks consistency. Please re-phrase it. Also, you have mentioned that only 10% of the costs of these activities were considered important for PSBI management….We included 20% of these costs as PSBI treatment and management, and calculated the human resource cost for this intervention based on the time spent by providers. It is not clear or needs clarification.
6. **Link between CHWs and nurses (Intervention 2.0):** Who were the service providers in this context? Make it clear because within the text at some area you mentioned nurse and other site CHEWs.
7. **Outpatient treatment (Intervention 4.0):**
i. Please elaborate the randomization process. How you avoided bias? Hint: blinding issue for RCT studies is very important for better result. Therefore, please provide clear and detailed description on randomization, the data collection process and analysis.
8. **Please write the methodology section in past tense.**

**Data**

1. What type of tool was used to collect the data?
2. What type of data source was you used? On the abstract part, it was mentioned that you used data from the African Neonatal Sepsis Trial (AFRINEST), conducted at five sites in three countries. Is that from secondary data source?
3. Who were the data collectors? How was data collected?
4. I have understood that the data were collected simultaneously during the intervention period. Therefore, do not you think that table 1 &2 were findings of the study that leads you to meet the objectives?
9. How you processed and analyzed the data? Please clearly elaborate the way you cleaned, processed or analyzed data.
10. In addition, specify any measurements for the outcome variables presented on result section.

**Result section**

1. Please add number of young infants covered, treated and outcome assessment with respect to their age and randomization to each regimen.
2. There were discrepancies on some of results presented on the table with description in the text.
3. Please put table 5 next to table 4, and provide descriptions on it.

**Discussion**

1. Please put your pertinent cost and cost-effectiveness-related findings on the first paragraph, and proceed discussing the findings.
2. There are some discrepancies between what was presented on the tables (result section) and what you were discussed. Please revise it.
3. In addition, you discussed with the 95% CI, which was good. But, you did not report it on the result section. This also needs revision.
4. The last two paragraphs of discussion part were not go with your objectives and results. Basically, as a general science, you are right, but consistent with your result. To discuss like this, you might undergone some advanced statistical analysis.

**Implication and conclusions**

1. The first two sentences support your results and what you were discussed. But, precede conclusions done on costs of treatment regimens and then, proceed with cost-effectiveness.
2. The rest of the sentences might not directly support your findings. How you related those activities with the neonatal survival without supporting findings? Hint: think of your objectives; cost and cost-effectiveness of PSBI regimens.
3. What are your recommendations based on the findings?

Other issues that need to be included

1. List of abbreviations: - there are a lot of abbreviations used in this manuscript.
2. Declarations
i. Availability data and material
ii. Author’s contributions
iii. Competing interests
iv. Consent for publication
v. Funding

**References:** Too old references were used which might be difficult to compare and contrast their findings to these study findings. For example, reference number 8 and 9.
